# Azomycin produced by *Pseudomonas* has both phytotoxic and anti-oomycete activity

**DOI:** 10.1128/jb.00292-25

**Published:** 2025-11-05

**Authors:** Zayda P. Morales Moreira, Kareena Kak, Zi-Wang Wei, Daniela Yanez-Ortuno, Nicole R. Wang, Jason B. Hedges, Melissa Y. Chen, Quentin Geissmann, Wentao Zhang, Syama Chatterton, Katherine S. Ryan, Cara H. Haney

**Affiliations:** 1 Department of Microbiology and Immunology, The University of British Columbia 8166, Vancouver, British Columbia, Canada; 2 Department of Chemistry, The University of British Columbia 8166, Vancouver, British Columbia, Canada; 3 Center for Quantitative Genetics and Genomics, Aarhus University 1006, Aarhus, Denmark; 4 Aquatic and Crop Resource Development, National Research Council Canada 85071 , Saskatoon, Saskatchewan, Canada; 5 Agriculture and Agri-Food Canada, Lethbridge Research and Development Centre 98670 , Lethbridge, Alberta, Canada; 6 Department of Biological Sciences, The University of Pittsburgh 171653, Pittsburgh, Pennsylvania, USA; Geisel School of Medicine at Dartmouth, Hanover, New Hampshire, USA

**Keywords:** root rot, *Aphanomyces euteiches*, nitroimidazole antibiotics, *Pisum sativum*

## Abstract

Members of the genus *Pseudomonas* synthesize diverse natural products that contribute to their versatility in free-living and host-associated lifestyles. Here, we characterized the *in vivo* functions of the nitroimidazole antibiotic azomycin in the genus *Pseudomonas*. We found that genes with similarity to azomycin biosynthesis genes *rohPQRST* are prevalent within the *Pseudomonas syringae* species complex and rarely present in the *Pseudomonas fluorescens* species complex. Azomycin production was detectable in culture by biocontrol strains *Pseudomonas* spp. DF41 and CMR5a. *Pseudomonas* sp. DF41 exhibited anti-oomycete activity that was lost in a *∆rohPQRST* mutant. Purified azomycin was sufficient to kill the oomycete pathogens *Aphanomyces* and *Phytophthora*. Although DF41 has been studied for its role in biocontrol of plant pathogens, we found that azomycin exhibited phytotoxicity against *Pisum sativum* (pea) plants at similar concentrations to those that inhibited oomycetes. However, consistent with its use as a biocontrol agent, *Pseudomonas* sp. DF41 only produced azomycin *in planta* when pea plants were infected with the oomycete pathogen *Aphanomyces euteiches*. Our findings suggest dual roles for azomycin in *Pseudomonas,* functioning both as a biocontrol agent of oomycete pathogens, as well as a phytotoxic molecule with a potential role in plant virulence.

**IMPORTANCE:**

While many natural products are studied for their roles in the treatment of plant or human disease, the ecological functions of natural products are understudied. We found that an antibiotic, azomycin, is produced by *Pseudomonas* species and has toxicity against both plants and oomycete pathogens. Our findings suggest a complex ecological role of azomycin production by *Pseudomonas* in both the amelioration and exacerbation of plant disease.

## INTRODUCTION

Bacteria in the genus *Pseudomonas* are metabolically diverse and produce a wealth of natural products. Many of these are well characterized for biocontrol activity against plant pathogens, including the antifungal 2,4-diacetylphloroglucinol, diverse lipopeptide toxins, siderophores, and hydrogen cyanide (1). Because these molecules were discovered in the context of mechanisms that contribute to the biocontrol activity of *Pseudomonas* spp., the ecological implications of their biosynthesis are clear. However, many other natural products, including those produced by *Pseudomonas,* are studied for their antimicrobial or other medicinal properties *ex vivo* (2); consequently, *in vivo* ecological functions of natural products are often unknown.

*Pseudomonas* strains produce diverse nitroaromatic compounds, including pyrrolnitrin (3) and azomycin (4). Nitroimidazoles, including azomycin, are a subgroup of nitroaromatic compounds that have broad-spectrum antimicrobial activity. They are choice antibiotics for anaerobic infections where, upon crossing the bacterial membrane, nitroimidazoles are reduced by bacterial ferredoxin to produce nitrogen radicals that cause bacterial DNA damage (5). However, nitroimidazoles can also be reduced by a number of biological enzymes, and thus can be effective treatments for diverse pathogens, including protozoans (5). Presumably, *Pseudomonas* produces azomycin as an antibiotic to compete with other microbes, but its non-specific mode of action suggests it may have diverse targets.

Although azomycin was initially described in the 1950s in *Nocardia mesenterica* (6) and *Streptomyces eurocidicus* (7), the genes that encode the underlying biosynthetic enzymes were not described until more than 60 years later (8). A five-gene biosynthetic cluster, *rohPQRST,* is responsible for azomycin production in *S. eurocidicus*, with highly similar genes in *Pseudomonas* spp. (8). The discovery of the genes that encode azomycin biosynthesis provides an opportunity to use a reverse genetics approach to link azomycin production to its ecological function.

In this study, we sought to identify the distribution of the *rohPQRST* cluster across the *Pseudomonas* genus and elucidate the ecological role of azomycin. We found that genes with similarity to azomycin biosynthesis genes are found in diverse members of the genus *Pseudomonas,* although primarily in plant pathogenic *Pseudomonas syringae*. We found that while two tested *P. syringae* strains did not produce azomycin during planktonic growth, two members of the *Pseudomonas fluorescens* species complex, including the biocontrol strain *Pseudomonas* sp. DF41 produced detectable azomycin. We therefore sought to characterize the ecological role of azomycin in the biocontrol strain *Pseudomonas* sp. DF41.

## RESULTS

### Non-pathogenic *Pseudomonas* produce the nitroimidazole antibiotic azomycin

In *S. eurocidicus* ATCC 27428*,* the nitroimidazole antibiotic azomycin is synthesized by enzymes encoded by a five-gene biosynthetic cluster *rohPQRST* (8). A gene cluster with similarity was also observed in *Pseudomonas* spp. (8). To determine how prevalent the *rohPQRST*-like gene cluster is in the *Pseudomonas* genus, we performed comparative genomics using the predicted amino acid sequences from *S. eurocidicus* RohPQRST (WP_102919045–WP_102919041) enzymes as query sequences (9). We first identified similar genes using Diamond BLASTP against a curated database (pseudomonas.com). We found the predicted *Pseudomonas* protein with the highest similarity to *S. eurocidicus* RohP was *Pseudomonas* sp. CMR5c C4K40_RS25395 with 54.6% amino acid identity. Like the *S. eurocidicus rohP* gene, the gene in CMR5c was found in a 5-gene predicted operon (C4K40_RS25395-75), and adjacent genes had 38.2%, 36.1%, 40.6%, 38.8% (*e* < 10^−12^) identity to *S. eurocidicus* RohQRST, suggesting these genes may encode enzymes that synthesize azomycin.

**Fig 1:**
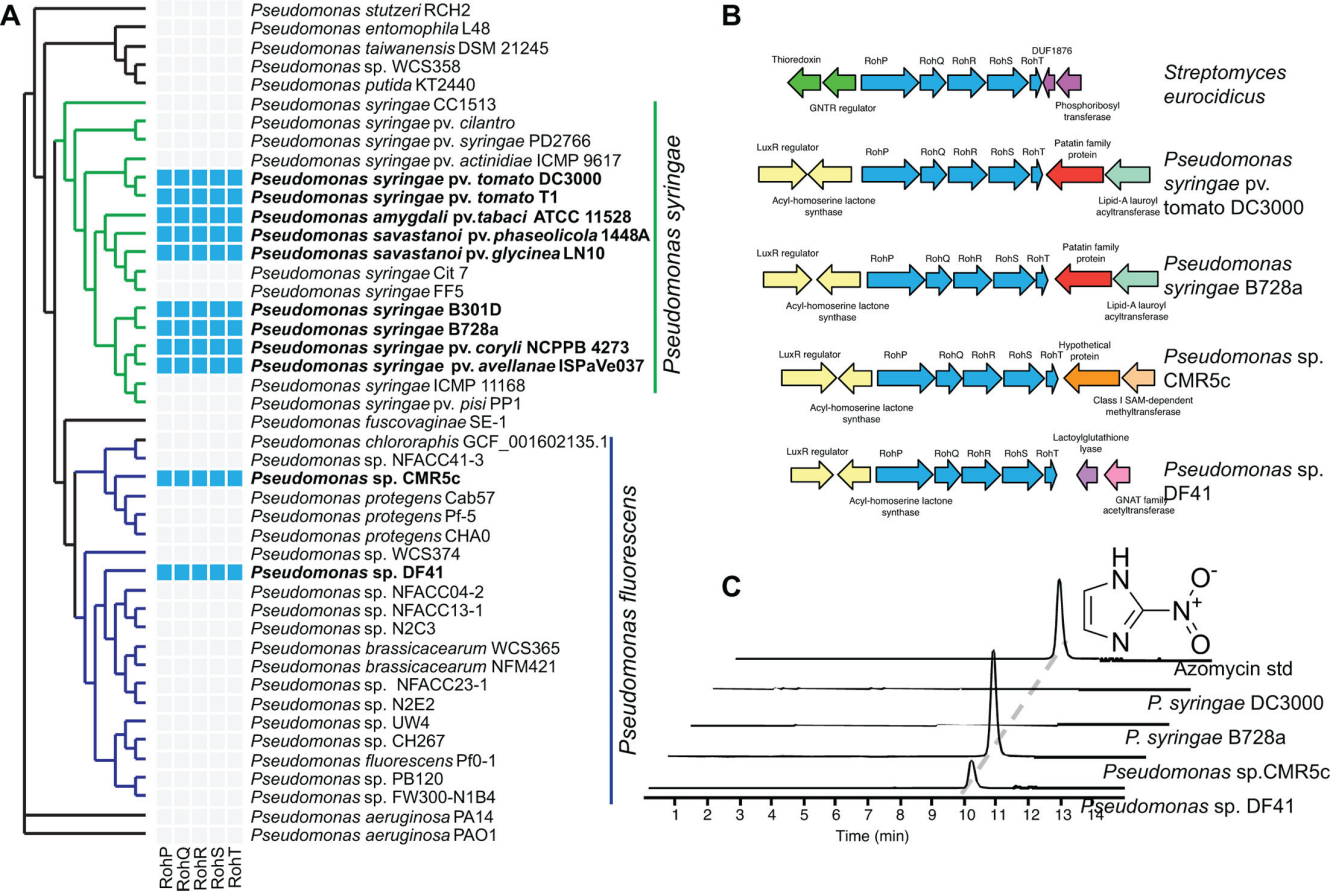
Non-pathogenic *Pseudomonas* produce the nitroimidazole antibiotic azomycin. (**A**) The *S. eurocidicus* RohPQRST (GenBank accession WP_102919045–WP_102919041) predicted amino acid sequences were used to identify the *rohPQRST* operon in *Pseudomonas* sp. CMR5c. The *S. eurocidicus* or CMR5c genes (Pseudomonas.com accession C4K40_RS25395–C4K40_RS25375) were then used with a comparative genomics tool for ortholog identification to identify RohPQRST-like proteins (blue squares) in diverse *Pseudomonas*. From a database of more than 3,800 genomes, 165 strains with the operon, including 146/383 (38%) strains from the *P. syringae* species complex (green portion of the tree) and 14/614 (2.3%) from the *P. fluorescens* species complex (blue portion of the tree) were identified (Fig. S1). A subset of strains is shown. (**B**) The *rohPQRST* operon is adjacent to genes encoding LuxRI in diverse *Pseudomonas*. Predicted protein reference numbers from GenBank (*S. eurocidicus*) or Pseudomonas.com (*Pseudomonas* species) are as follows: *S. eurocidicus* WP_102919047–WP_102919039*; P. syringae* pv. tomato DC3000 P0L91_RS07075–P0L91_RS07035, *P. syringae* B728a Psyr_1622–Psyr_1614, CMR5c genes C4K40_RS25405–C4K40_RS25465 and *Pseudomonas* sp. DF41 CD58_RS05015–CD58_RS05055. (**C**) *Pseudomonas* sp. CMR5c and *Pseudomonas* sp. DF41 produces detectable azomycin by liquid chromatography-high resolution mass spectrometry (LC-HR-MS analysis extracted ion chromatogram, EIC; *m/z* 114.0298) when grown in lysogeny broth (LB) medium at 28°C for 5 days. Bacterial samples were concentrated 100-fold. Chromatograms are staggered for clarity, and a 50 µM azomycin standard was used.

To identify similar genes across the genus using a non-redundant genomic database, we used the *S. eurocidicus* or *Pseudomonas* sp. CMR5c RohPQRST-like amino acid sequences to query a previously described non-redundant database of more than 3,800 *Pseudomonas* genomes (9, 10). Using both queries, we found genes that are predicted homologs of *rohPQRST* in 165 *Pseudomonas* strains of the genomes queried (4.2%). This includes 146 of the 383 (38%) of the genomes from *P. syringae* species complex in the database (Fig. S1), including model strains *P. syringae* pv. *tomato* DC3000 and *P. syringae* B728a (Fig. 1A). We also identified *rohPQRST*-like genes in 14 of 614 (2.3%) of the genomes from the *P. fluorescens* species complex (Fig. S1), including the beneficial strains *Pseudomonas* sp. CMR5c and *Pseudomonas* sp. DF41 (Fig. 1A). Of the remaining nearly 2,900 genomes, we only identified the putative operon in five additional genomes (Fig. S1). These results indicate that *rohPQRST* homologs are common in *P. syringae* and are rarely present in other members of the genus *Pseudomonas*.

To determine if the genetic context of the RohPQRST operon was similar across strains, which would support a common acquisition event, we explored the genomic context of the *rohPQRST* gene cluster. In all cases, the *Pseudomonas rohPQRST* gene clusters were located adjacent to *luxRI* quorum-sensing biosynthesis and sensor genes, suggesting the possible regulation of *rohPQRST* expression by quorum signaling. However, the genes on the other side of the operon were variable across strains (Fig. 1B). These findings indicate that there are likely multiple acquisition events of the *rohPQRST* genes in *Pseudomonas* spp.

To determine if the presence of the *rohPQRST* genes cluster results in azomycin biosynthesis in *Pseudomonas* spp., we used liquid chromatography-high-resolution mass spectrometry (LC-HR-MS) analysis, in two plant pathogens in the *P. syringae* species complex (*P. syringae* pv. *tomato* DC3000 and *P. syringae* B728a) and two beneficial strains in the *P. fluorescens* species complex (*Pseudomonas* sp. CMR5c and *Pseudomonas* sp. DF41). Azomycin was detected only in the supernatant of the two beneficial strains, *Pseudomonas* sp. CMR5c and *Pseudomonas* sp. DF41 after 5 days of growth in LB broth (Fig. 1C). This result is consistent with previous reports that some *Pseudomonas* strains can produce azomycin (4).

**Fig 2:**
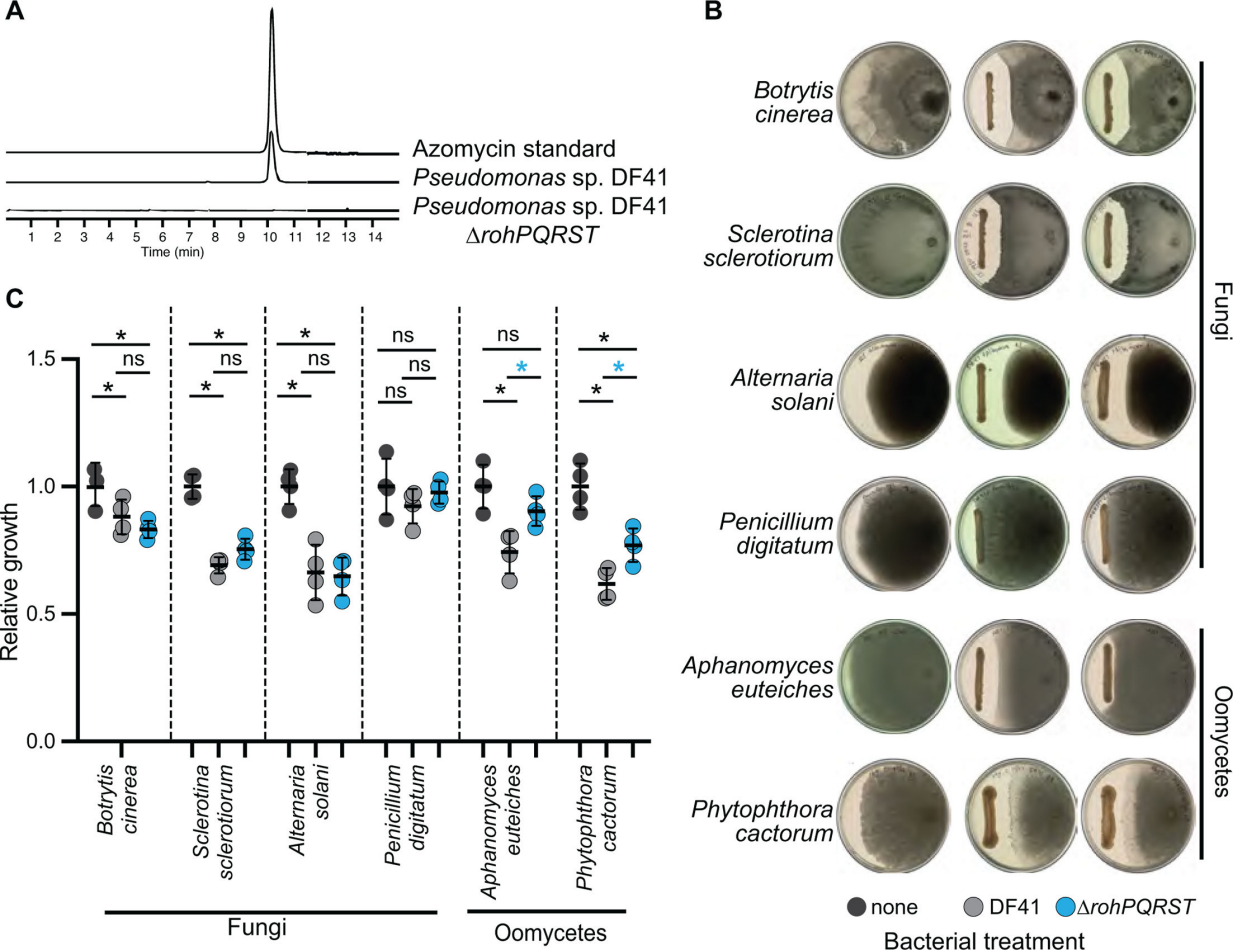
*Pseudomonas* sp. DF41 inhibits oomycete pathogens in an azomycin-dependent manner. (**A**) LC-HR-MS analysis (extracted ion chromatogram, EIC; *m/z* 114.0298) *Pseudomonas* sp. DF41, the DF41 ∆*rohPQRST* mutant, and a 50 µM azomycin standard. (**B**) Fungal and oomycete plant pathogens were grown on potato dextrose agar (PDA) in the presence or absence of *Pseudomonas* sp. DF41 or the ∆*rohPQRST* mutant. Representative images are shown. (**C**) Relative growth of fungal or oomycete pathogens shown in B was calculated by dividing growth on each plate by the mean growth of no bacterial control plates; *P < 0.05 by analysis of variance (ANOVA) and Tukey’s HSD; bars indicate means ± SD. ns, not significant; *n* = 4.

To confirm that the *rohPQRST* locus in *Pseudomonas* encodes enzymes necessary for the detected biosynthesis of azomycin, we deleted the *rohPQRST* operon in the beneficial biocontrol strain *Pseudomonas* sp. DF41. Through LC-HR-MS, we confirmed that the DF41 ∆*rohPQRST* mutant could not produce azomycin (Fig. 2A). These data indicate that, like in *Streptomyces* spp., the *rohPQRST* operon in *Pseudomonas* is necessary for azomycin biosynthesis.

### Azomycin inhibits the growth of oomycete pathogens and pea plants

*Pseudomonas* sp. DF41 is a well-studied biocontrol strain in the *brassicacearum/mediterranea/corrugata* subclade of the *P. fluorescens* species complex. DF41 has broad antimicrobial activity against fungal (11) and oomycete (12) plant pathogens. DF41 produces the non-ribosomal lipopeptide toxin sclerosin, which has genetic and structural similarity to syringomycin and syringopeptin from *P. syringae* (11). Interestingly, mutants that do not produce sclerosin still retain some activity against fungal pathogens (11), and purified sclerosin has no activity against zoospores of the oomycete pathogen *Phytophthora* (13), suggesting that additional antifungal and anti-oomycete compounds are produced by DF41. While primarily studied for its antibacterial activity, the mode of action of azomycin and other nitroimidazole antibiotics is fairly non-specific (5). Although they are primarily toxic against bacteria, nitroimidazole compounds also have been described as toxic against fungi and other eukaryotes (5, 14). We therefore hypothesized that azomycin may have activity against fungi and oomycetes.

To test whether azomycin has activity against oomycete and fungal plant pathogens, we tested DF41 and the *∆rohPQRST* mutants for their ability to inhibit growth *in vitro*. As previously reported, we found that DF41 inhibited the growth of fungal and oomycete pathogens (11) (Fig. 2B and C). We found that DF41 exhibited azomycin-dependent inhibition of the growth of two oomycete plant pathogens *Aphanomyces euteiches* AE1 (causative agent of root rot in peas [15]) and *Phytophthora cactorum* FF42 (a root rot pathogen of larch [16]) (Fig. 2B and C). We observed a significant reduction in the growth of both pathogens by DF41, which was significantly diminished, although not completely lost, in the *∆rohPQRST* mutant (Fig. 2B and C), suggesting that azomycin, combined with other antimicrobials produced by DF41, inhibits oomycetes. We also found that DF41 inhibited the growth of the fungal pathogens *Alternaria solani* P1652 A17*, Sclerotinia sclerotiorum* 1980*,* and *Botrytis cinerea* BO5.10 (Fig. 2B and C). However, the inhibition was azomycin-independent as the *∆rohPQRST* mutant still inhibited the growth of these fungal pathogens to a similar degree as wild-type DF41 (Fig. 2B and C). *Penicillium digitatum* was not significantly inhibited by DF41 or the DF41 *∆rohPQRST* mutant (Fig. 2B and C). Collectively, these findings indicate that azomycin has activity against phylogenetically diverse oomycetes, but not fungi.

**Fig 3:**
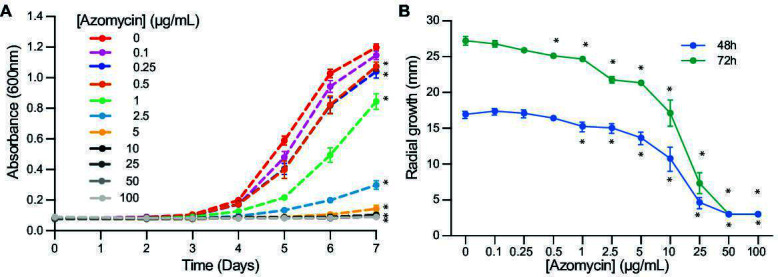
Purified azomycin inhibits *A. euteiches* AE1 growth *in vitro*. (**A**) 5 × 10^3^ AE1 zoospores were inoculated into 48-well plates containing PDB with a dilution series of azomycin. Growth was quantified over 7 days using a plate reader and measuring absorbance at OD_600_. *n* = 3 plates with five wells per replicate. Bars indicate means ± SD. (**B**) Five millimeter AE1 plugs were placed in the center of Petri dishes containing PDA with a dilution series of azomycin. *n* = 3 with three plates per replicate. Bars indicate means ± SD. *P < 0.05 by ANOVA and Tukey’s HSD relative to the no azomycin control.

We tested whether purified azomycin could inhibit *A. euteiches* AE1(15) using a dilution series ranging from 0.1 to 100 µg/mL in both liquid potato dextrose broth (PDB) and solid potato dextrose agar (PDA). We tested both solid and liquid media since the rhizosphere is a mix of solid and liquid surfaces, and microbes may differ in their antibiotic susceptibility in different environments. In PDB, we found significant inhibition of *A. euteiches* AE1 with concentrations as low as 0.25 µg/mL and complete inhibition of growth at 5–10 µg/mL (Fig. 3A). We found similar results on PDA with significant inhibition of AE1 with concentrations as low as 0.5–1 µg/mL and with complete inhibition of growth at 50–100 µg/mL (Fig. 3B). These findings indicate that azomycin is sufficient to inhibit the growth of *A. euteiches* AE1.

**Fig 4:**
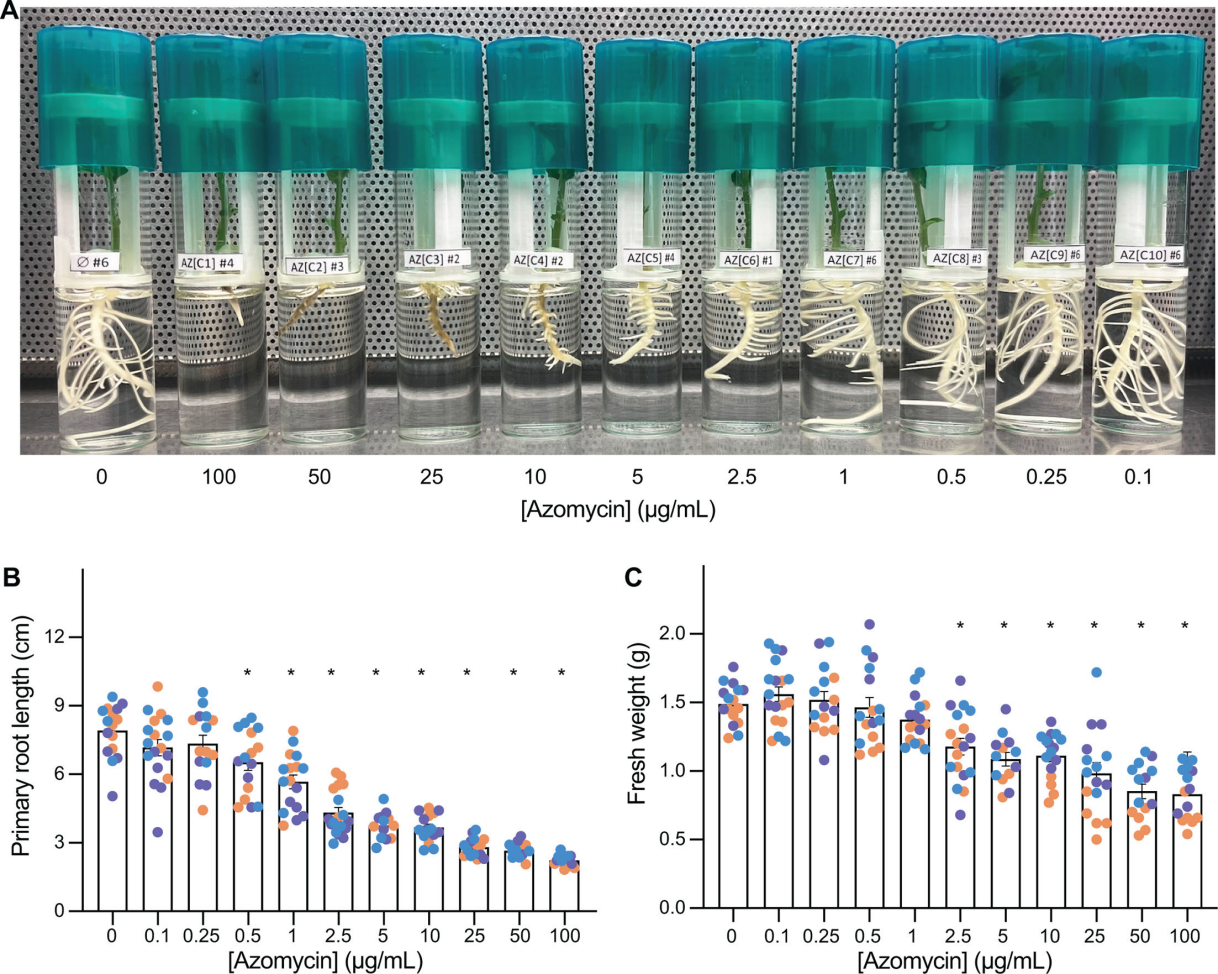
Azomycin is phytotoxic to *P. sativum* (pea) plants. (**A**) Individual pea plants were germinated and grown under gnotobiotic hydroponic conditions in B5 media with a concentration gradient of azomycin. (**B**) Root length and (**C**) plant weight were measured after 7 days. *n* = with 3–6 plants per replicate with different colors representing different replicates. The bars indicate means ± SD; *P < 0.05 by ANOVA and Tukey’s HSD relative to the no azomycin control.

As oomycete pathogens of plants are notoriously difficult to control, we sought to explore the potential of azomycin as a biocontrol treatment for oomycete pathogens. However, oomycetes are closely related to plants and thus are often susceptible to overlapping toxins. Furthermore, nitroimidazoles have been reported to have toxicity on plants (17). *A. euteiches* is an important causative agent of root rot of *Pisum sativum* (pea); to assay the effects of *A. euteiches*, *Pseudomonas* sp. DF41 and azomycin treatment of pea plants, we developed a custom 3D-printed pea growth system where a 3D printed “pea holder” can be mounted in an autoclavable flat-bottom glass tube (Fig. S2). Pea seeds can then be surface-sterilized and grown gnotobiotically. We tested the same azomycin dilution series as we tested for *A. euteiches* AE1 on *P. sativum* L. cultivar CDC Meadow plants grown in Gamborg′s B-5 basal medium. We observed significant stunting of plant root growth at 0.5 µg/mL azomycin, and a significant reduction in plant weight at 2.5 µg/mL (Fig. 4). As these are similar concentrations to those we found to inhibit *A. euteiches* AE1 (Fig. 3), our findings indicate that concentrations of the antibiotic that are sufficient to control *A. euteiches* may also be harmful for plants.

### Azomycin is only produced *in planta* in the presence of a pathogen

**Fig 5:**
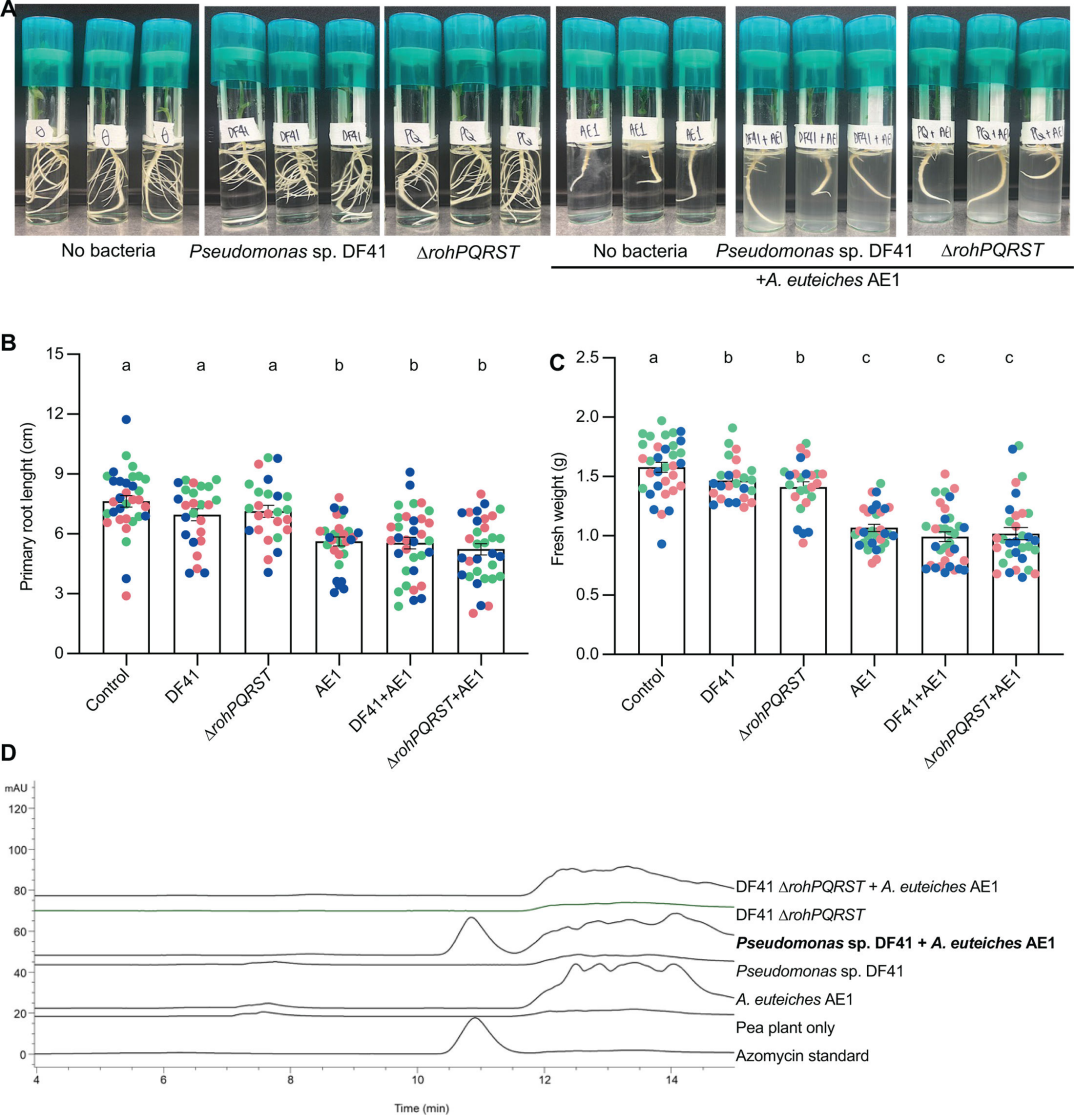
Azomycin is produced in the presence of oomycetes, but not in the presence of plants alone. (**A**) Pea plants were grown hydroponically and treated with *Pseudomonas* sp. DF41 or the DF41 ∆*rohPQRST* mutant (to a final OD_600_ of 0.0005) with or without 5 × 10^3^ spores of *A. euteiches* AE1. (**B, C**) Quantification of plant root length (**B**) and fresh weight (**C**) of the plants shown in A. Letters indicate *P* < 0.05 by ANOVA and Tukey’s HSD; bars indicate mean ± SD; each dot represents a plant (*n* = 12–18 with 4–6 plants from each of three biological replicates) with different colors representing different replicates. (**D**) High-performance liquid chromatography (HPLC) (detection wavelength λ = 330 nm) analysis of pea root exudates with or without bacterial or *A. euteiches* treatment shows that DF41 produces azomycin in the presence of *A. euteiches* AE1, but not in pea plants alone. A 50 µM azomycin standard was used for reference.

*Pseudomonas* sp. DF41 is effective at controlling fungal pathogens in plants (11), suggesting that azomycin *in planta* may not cause significant issues for plant growth. To test if DF41 stunted plants in an azomycin-dependent manner, we tested wild-type and the DF41 *∆rohPQRST* mutant on pea plants. We found that treatment of pea seedlings with DF41 did not affect plant root length and caused a slight but significant decrease in plant fresh weight (Fig. 5A through C). However, the decrease in plant fresh weight was not azomycin dependent (Fig. 5A through C).

Because purified azomycin is phytotoxic, but DF41 is not, we hypothesized that DF41 might not produce azomycin in the rhizosphere. Azomycin is synthesized from the precursor arginine, which is present at low levels in the rhizosphere (18). Indeed, high-performance liquid chromatography (HPLC) analysis confirmed that the levels of azomycin produced by DF41 in the pea rhizosphere are below the detection limit (Fig. 5D). The finding that the phytotoxin azomycin is not produced by DF41 *in planta* is consistent with the use of DF41 as a biocontrol strain. It suggests that DF41 might not produce azomycin due to limited bacterial growth in the rhizosphere, insufficient precursor availability, or lack of *rohPQRST* gene induction.

Since DF41 can kill *A. euteiches in vitro*, we hypothesized that azomycin might be produced in the plant rhizosphere in the presence of *A. euteiches*. While azomycin production might result in decreased levels of the *A. euteiches* pathogen and a decrease in disease, it is similarly possible that azomycin will also cause damage to the plants directly, resulting in enhanced disease. We treated pea plants with *Pseudomonas* sp. DF41 and the azomycin-deficient *ΔrohPQRST* mutant with *A. euteiches* AE1. We found that the presence of *A. euteiches* AE1 resulted in smaller plants and shorter roots, but this was not changed by the presence of DF41 or the *ΔrohPQRST* mutant. We found by HPLC analysis that azomycin was produced by DF41 in the pea rhizosphere in the presence of *A. euteiches* AE1 (Fig. 5D). These results suggest that the negative effects of azomycin on pea plants may counteract the benefit from reduced pathogen growth under laboratory conditions.

## DISCUSSION

While nitroimidazole antibiotics are well known for their role in treating anaerobic bacterial infections (5), we sought to characterize their ecological role *in vivo*. The recent discovery of azomycin biosynthesis genes *rohPQRST* (8) in *Streptomyces* allowed us to use a genetic approach to characterize production in *Pseudomonas* and its role *in vivo*. We found that azomycin has toxicity against both plants and oomycetes, underscoring a potentially complex ecological role. Some anti-oomycete compounds can also exhibit dual toxicity against plants and oomycetes, while others are relatively specific to oomycetes (19). Collectively, this work highlights the need to understand the consequences of natural product biosynthesis in the context of both the host and the microbiome.

Root rot is a complex disease that is the result of the interaction of many biotic agents, including *Aphanomyces, Pythium,* and *Fusarium* (20). It is tempting to speculate that the production of phytotoxic azomycin by the root-associated *Pseudomonas* sp. DF41 in the presence of *Aphanomyces* could exacerbate plant disease. Alternatively, if azomycin inhibits the spread of oomycete pathogens, the plant community might benefit even if there are negative impacts on individual plants. While we did not find direct evidence for either hypothesis in a highly controlled lab environment, it is difficult to know whether azomycin production by *Pseudomonas* spp. would ameliorate or exacerbate root rot disease in the field. That a single bacterial-derived natural product affects a plant and an oomycete pathogen underscores the challenges of predicting disease outcomes in a highly complex field environment.

Although we found the *rohPQRST* gene cluster in diverse plant pathogenic *P. syringae* strains, we did not detect azomycin production *in vitro* in either of the two plant pathogens tested, *P. syringae* pv. tomato DC3000 or *P. syringae* B728a. While it is possible that other *P. syringae* strains make azomycin *in vitro*, there may be an *in planta* or other signal required for gene induction in these two strains and subsequent azomycin biosynthesis. Given the observed phytotoxic activity of azomycin, and the prevalence of the operon in *P. syringae* strains, it would be interesting to test whether the *P. syringae rohPQRST* genes are induced *in planta,* and whether the genes contribute to virulence.

Root rot diseases are complex and notoriously difficult to control as they are the result of complex interactions of multiple pathogens (20). Biocontrol has been an appealing approach to controlling these complex diseases, particularly for those with limited sources of plant genetic resistance (21). However, the findings that a compound that can control oomycete pathogens *in vitro* also has phytotoxicity underscore the complexity of these interactions. Further work understanding the dynamics of azomycin production in the field and *in planta* will reveal whether this molecule can exacerbate or ameliorate plant disease.

## MATERIALS AND METHODS

### Bacterial, fungal, and oomycete growth

Bacteria, fungi, and oomycetes used in this study are summarized in Table S1. Bacterial and mutant strains were routinely cultured in LB overnight at 28°C with shaking at 180 rpm; except for *Escherichia coli* strains, which were cultured at 37°C. Fungal and oomycete strains were routinely grown in PDA at room temperature (23°C).

### Comparative genomics and phylogenetic tree

The *rohPQRST* gene cluster, originally characterized in *Streptomyces,* was previously found to be present in *Pseudomonas* spp. (8). The RohPQRST amino acid sequences from *Streptomyces* were used as query sequences to plot the presence and distribution of the *rohPQRST* cluster in the *Pseudomonas* genus using the comparative genomics analyses performed in the platform PyParanoid (9). Briefly, using a training data set of high-quality *Pseudomonas* genomes, PyParanoid was previously used to generate a pangenome using conventional similarity clustering methods (9). This allows the comparison of genomes of individual strains to this pangenome to determine homolog group presence-absence data. The database includes the presence and absence of 24,066 discrete homology groups to 3,894 diverse genomes from strains across the *Pseudomonas* genus and assigns homology group membership to 94.2% of the 22.6 million protein sequences in our combined database. Phylogenetic trees were generated using concatenated 122 core gene sequences from *Pseudomonas* as previously described and generated with FastTree (9). The genomic context of azomycin-producing bacteria was explored in The *Pseudomonas* Genome Database (https://www.pseudomonas.com/) with support of NCBI (https://www.ncbi.nlm.nih.gov).

### Deletion of the azomycin cluster in *Pseudomonas* sp. DF41

The *rohPQRST* gene cluster was deleted from *Pseudomonas* sp. DF41 uses two-step allelic exchange (22). Briefly, 700–800 bp regions flanking either side of the *rohPQRST* cluster were PCR-amplified, joined through overlap PCR, cloned into the pEXG2 suicide vector, and then transformed into *E. coli* DH5ɑ. The plasmid was verified through PCR and Sanger sequencing before being transformed into the donor strain *E. coli* SM10λpir. The deletion plasmid was introduced into DF41 through conjugation, by mixing a 1:2 ratio of SM10λpir donor:DF41 recipient, then plating them in mating spots on King’s B agar for 2–4 h at 30°C. Mating spots were scraped off and resuspended in 100 mM MgSO_4,_ and then transconjugants that underwent a single crossover event to integrate the plasmid into the chromosome were selected for on gentamicin 25 and 15 µg/mL of nalidixic acid. The transconjugants were grown in LB overnight without selection and then plated on no salt LB with 10% sucrose to select for cells that had undergone a second recombination event to excise the plasmid backbone. Successful mutants were screened for gentamicin sensitivity, then verified by PCR and Sanger sequencing.

### Detection of azomycin production by members of the *Pseudomonas* genus

*Pseudomonas* sp. DF41, *Pseudomonas* sp. DF41 *ΔrohPQRST*, *P. syringae* pv. *tomato* DC3000, *P. syringae* B728a, and *Pseudomonas* sp. CMR5c were grown separately in 50 mL of LB medium in a 250 mL flask at 28°C, with shaking at 180 rpm for 5 days. The cultures were transferred to a 50 mL Falcon tube and centrifuged (4°C, 5,000 rpm, 10 min) for azomycin extraction (8). The supernatant was acidified with HCl to pH 1–2, and then extracted twice with an equal volume of ethyl acetate (EtOAc). The EtOAc portions were combined and concentrated under reduced pressure. The crude was resuspended in DMSO (or a mixture of DMSO and ACN) and centrifuged at 12,000 rpm for 5 min. The resultant supernatant was subjected to HPLC or LC-HR-MS analysis. HPLC analysis was carried out using a Luna C18(2), 5 µm, 4.6 mm ID × 250 mm column (Phenomenex), with the detection λ = 330/360 nm (detection wavelength/reference wavelength). Elution was performed at 0.5 mL/min with a mobile phase mixture consisting of a linear gradient of water and acetonitrile ([vol/vol]: 90:10, 0 to 5 min; 0:100, 6 to 10 min; 95:10, 11 to 15 min), both of which contain 0.01% (vol/vol) formic acid. For HR-MS analysis, extracted ion chromatograms (EICs; *m/z* 114.0298) were used for the detection of azomycin. LC/MS was performed on an Agilent 6546 Q-TOF using the Agilent 1260 Infinity II HPLC system. The same elution method was adapted as described above, using a C18(2) column, and extracted ion chromatograms (EICs; *m/z* 114.0298) were used for the detection of azomycin.

### Antagonism tests

To explore the role of azomycin, the azomycin-producer *Pseudomonas* sp. DF41 and its azomycin biosynthesis mutant *Pseudomonas* sp. DF41 *ΔrohPQRST* were tested against several fungal and oomycete strains (Table S1). For fungal and oomycete antagonism tests, we tested the fungal pathogens *S. sclerotiorum* 1980, *B. cinerea* BO5.10, *P. digitatum*, and the oomycete pathogens *A. euteiches* AE1 and *Phytophthora cactorum* FF42. A 5 mm fungal/oomycete plug was placed on one side of a PDA plate, and *Pseudomonas* sp. DF41 or *Pseudomonas* sp. DF41 *ΔrohPQRST* were single-streaked on the other side (23). Antagonism (fungal/oomycete growth inhibition) was evaluated after 5 days at room temperature (23°C). Relative growth was calculated by dividing the growth on each plate by the mean growth of the no bacterial control plates.

### *A. euteiches* AE1 growth in the presence of the antibiotic azomycin

*A. euteiches* AE1 was grown as previously described (24). Briefly, 20 mycelial plugs from the growing edge of 5-day-old *A. euteiches* cultures were placed in 40 mL of peptone-glucose broth (peptone 20 g/L and glucose 5 g/L). After 5 days, the broth was decanted and the plugs were washed with 50 mL of 50% mineral solution (CaCl_2_·2H_2_O 0.26 g/L + KCl 0.07 g/L + MgSO_4_·7H_2_O 0.49 g /L) twice, with 1.5 h intervals between washes. The last wash was discarded, and 30 mL of full-strength mineral solution was added. The plugs were incubated overnight at room temperature. After the incubation period, the solution was collected, and a 1 mL aliquot was pipetted into a glass test tube and vortexed for 30 s to allow zoospores to encyst. Zoospores were counted with a hemocytometer.

A concentration of 5 × 10^3^ spores of *A. euteiches* AE1 was added to 48-well plates containing 800 µL of PDB with 10 different concentrations (100, 50, 25, 10, 5, 2.5, 1, 0.5, 0.25, and 0.1 µg/mL) of azomycin (2-nitroimidazole) plus a control without the addition of azomycin. Plates were placed in a controlled environment chamber with a 16/8 h photoperiod and a temperature of 23°C/21°C day/night. *A. euteiches* AE1 growth was measured daily during 7 days using a plate reader (absorbance 600 nm) (SpectraMax). Three biological replicates with five technical replicates per concentration were performed.

The same azomycin concentrations were added to PDA and poured into 60 mm Petri dishes. Five millimeter *A*. *euteiches* AE1 plugs were placed in the center of the Petri dish, and the oomycete growth was measured after 2 and 3 days. Three biological replicates with three technical replicates per concentration were carried out.

### Pea seeds disinfection and germination

Seeds of *P. sativum* L. cultivar CDC Meadow were surface-disinfected (70% ethanol, 1 min; 2.5% sodium hypochlorite solution, 10 min; rinsed six times with sterile deionized water), and germinated in the dark in 0.8% PhytoAgar for 48 h at room temperature (23°C).

### *P. sativum* growth in the presence of the antibiotic azomycin

After 2 days of germination, *P. sativum* germinated seeds were placed in a custom 3D printed “pea holder” (surface sterilized by chlorine gas for 1 h) (Fig. S2) attached to a sterile flat-bottom glass tube (25 mm × 95 mm) containing 18 mL of Gamborg′s B-5 basal medium with minimal organics. Ten concentrations of azomycin (100, 50, 25, 10, 5, 2.5, 1, 0.5, 0.25, and 0.1 µg/mL) plus a control without addition of azomycin were added to the Gamborg′s B-5 media (Sigma) on the same day as seedling transfer. Pea plants were grown in a controlled environmental chamber with a 16/8 h photoperiod and a temperature of 23°C/21°C day/night. Fresh weight and primary root length were measured 7 days after transfer (9-day-old seedlings). Three biological replicates with 3–6 technical replicates per concentration were carried out. Only plants with roots growing through the central hole of the “pea holder” were used for analysis.

### Root rot (*A. euteiches*) control by the azomycin-producing *Pseudomonas* sp. DF41 in peas

*Pseudomonas* sp. DF41 and its azomycin mutant *ΔrohPQRST* were inoculated in pea plants with or without a causal agent of root rot, *A. euteiches* AE1. Pea plants were germinated and transferred to “pea holders” as described above. They were inoculated with *A. euteiches* and/or DF41 at the time of transfer (2-day-old seedlings). *A. euteiches* AE1 inoculum was prepared as described above, and spores were added at a concentration of 5 × 10^3^ spores of *A. euteiches* AE1 per plant. DF41 and the DF41 *∆rohPQRST* mutant were grown overnight, washed, and diluted to a final OD_600_ = 0.0005. We quantified plant weight, root length, and observed disease symptoms after 7 days of incubation with DF41 and the plants (9-day-old seedlings). In addition, we performed HPLC to quantify azomycin production.

## SUPPLEMENTAL MATERIAL

Supplemental figures and tables jb.00292-25-s0001.pdf Figures S1 and S2, and Tables S1.

ASM does not own the copyrights to Supplemental Material that may be linked to, or accessed through, an article. The authors have granted ASM a non-exclusive, world-wide license to publish the Supplemental Material files. Please contact the corresponding author directly for reuse.
