# Supplementary material for: Azomycin produced by Pseudomonas has both phytotoxic and anti-oomycete activity
Source: J Bacteriol. 2025 Nov 5;207(12):e00292-25. doi: 10.1128/jb.00292-25 (PMC12713392; doi:10.1128/jb.00292-25)
Supplement: Supplemental figures and tables — Figures S1 and S2, and Tables S1. [file jb.00292-25-s0001.pdf]

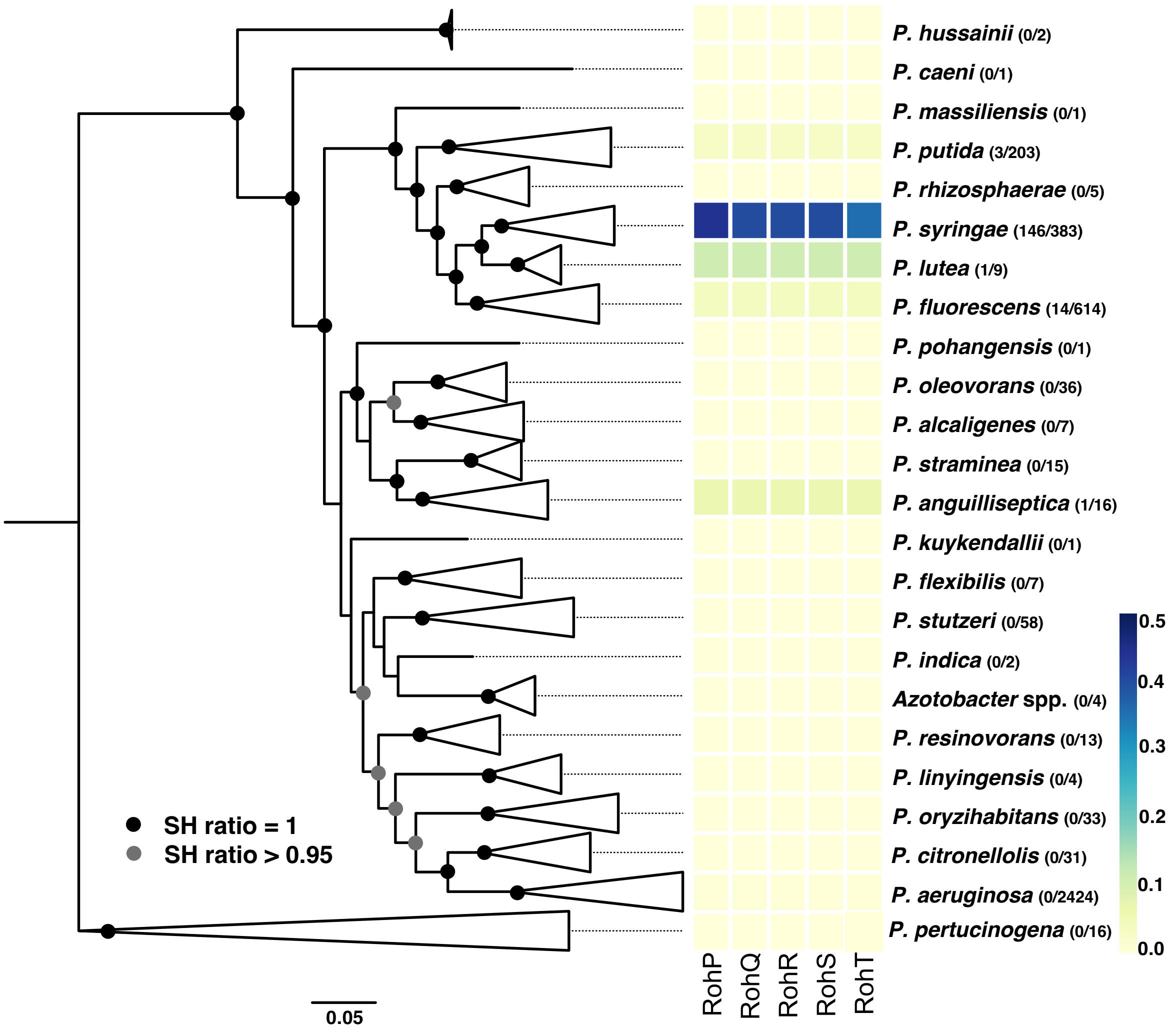

Figure S1. Distribution of predicted azomycin biosynthesis genes across the genus *Pseudomonas*. Using a database of 3894 Pseudomonas strains, and a query sequence of *S. eurocidicus* or *Pseudomonas* sp. CMR5c RohPQRST predicted amino acid sequences, we identified 165 strains with the predicted RohPQRST operon, including 146/383 (38%) strains from the *P. syringae* species complex (green portion of the tree) and 14/614 (2.3%) from the *P. fluorescens* species complex (blue portion of tree) were identified.

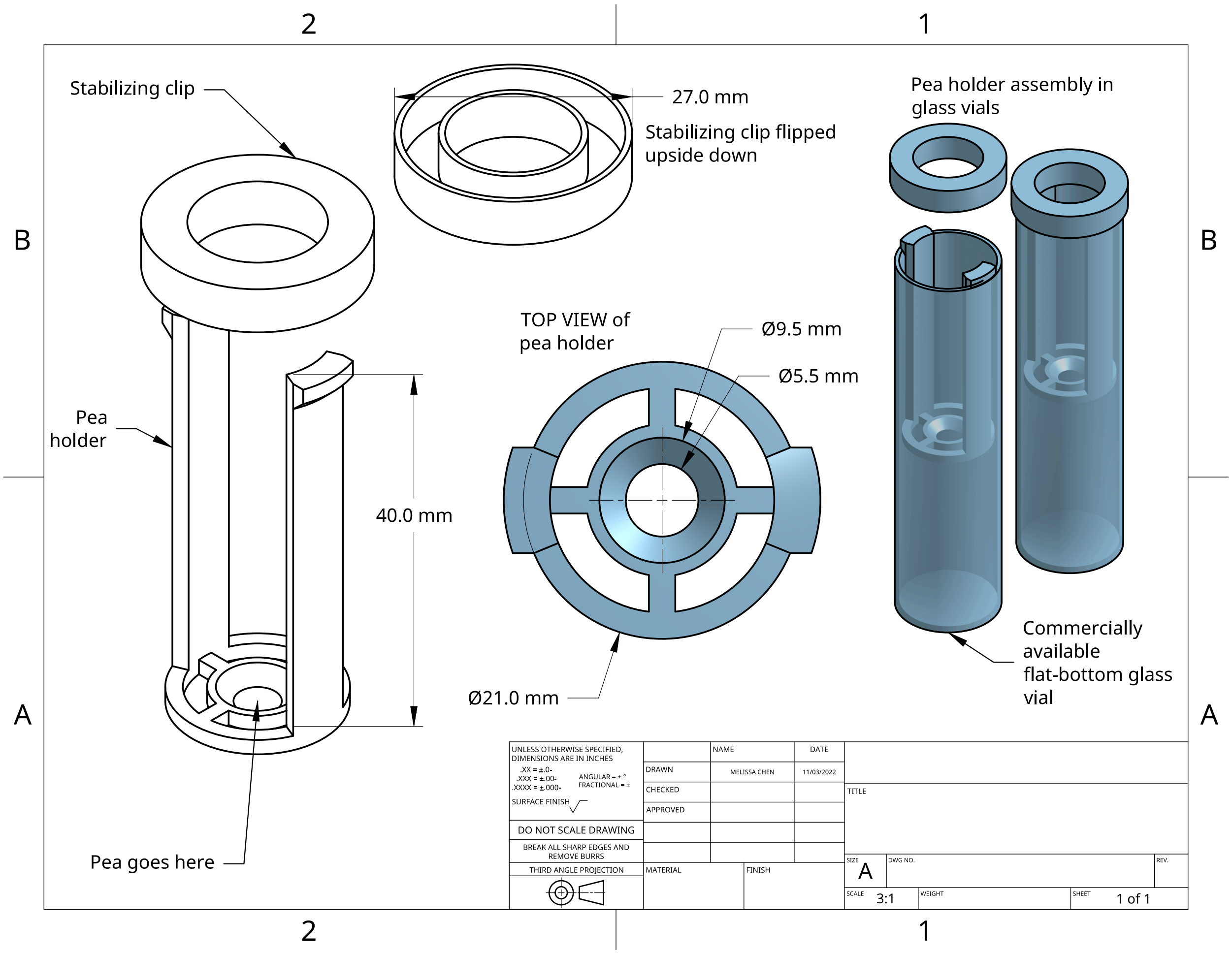

Figure S2. Schematic for 3D printed "pea holder". A 3D printed insert to fit inside a sterile 25mm x 95 mm glass-bottom tube. The holder hooks over the top of the tube, and has a support with a hole, allowing pea roots to grow into the liquid media below, and shoots to grow upward.

Table S1. Strains (A), mutants (B), and primers (C) used in this study

(A)

Strain Name	Microorganism	Use in this paper	Reference
Pseudomonas syringae pv. *tomato* DC3000	Bacterium	Azomycin production test	(Cuppels 1986)
Pseudomonas sp. DF41	Bacterium	Azomycin production test	(Savchuk and Dilantha Fernando 2004)
Pseudomonas syringae B728a	Bacterium	Azomycin production test	(Loper 1987)
Pseudomonas sp. CMR5c	Bacterium	Azomycin production test	(Perneel et al. 2007)
Sclerotinia sclerotiorum 1980	Fungus	Antagonism test	(Derbyshire et al. 2017)
Botrytis cinerea BO5.10	Fungus	Antagonism test	(Amselem et al. 2011)
Alternaria solani P1652 A17	Fungus	Antagonism test	(Edel Pérez López Lab)
Penicillium digitatum	Fungus	Antagonism test	(Edel Pérez López Lab)
Aphanomyces euteiches AE1	Oomycete	Antagonism test	(Sivachandra Kumar et al. 2021)
Phytophthora cactorum FF42	Oomycete	Antagonism test	(Dort and Hamelin 2024)

(B)

Mutant Name	Locus Tag	Predicted Function
DF41 *ΔPQRST*	CD58_RS05025<br>CD58_RS05030<br>CD58_RS05035<br>CD58_RS05040<br>CD58_RS05045	Azomycin Biosynthesis

(C)Primers

Primer Name	Sequence	Purpose
RohPQRST_SF	TCATTCACGCCATCTCCGTG	Sequencing
PacI_RohPQRST_F1-v2	catgTTAATTAACTACAAGGCTGTCAGCCTGG	Amplifying upstream homologous region
RohPQRST_R1	TGCGGAATACGAGGTGACCTCGCGCGCTAACGATTCAGTA	Amplifying upstream homologous region
RohPQRST_F2	TACTGAATCGTTAGCGCGCGAGGTCACCTCGTATTCCGCA	Amplifying downstream homologous region
BamHI_RohPQRST_R2	catgGGATCCAGGCCTGTATGCGGAACTTC	Amplifying downstream homologous region
RohPQRST_SR	GTCGCTATGACATTCCCGCA	Sequencing

Amselem, J., Cuomo, C. A., van Kan, J. A. L., Viaud, M., Benito, E. P., Couloux, A., Coutinho, P. M., de Vries, R. P., Dyer, P. S., Fillinger, S., Fournier, E., Gout, L., Hahn, M., Kohn, L., Lapalu, N., Plummer, K. M., Pradier, J. M., Quévillon, E., Sharon, A., Simon, A., Have, A., Tudzynski, B., Tudzynski, P., Wincker, P., Andrew, M., Anthouard, V., Beever, R. E., Beffa, R., Benoit, I., Bouzid, O., Brault, B., Chen, Z., Choquer, M., Collémare, J., Cotton, P., Danchin, E. G., Da Silva, C., Gautier, A., Giraud, C., Giraud, T., Gonzalez, C., Grossetete, S., Güldener, U., Henrissat, B., Howlett, B. J., Kodira, C., Kretschmer, M., Lappartient, A., Leroch, M., Levis, C., Mauceli, E., Neuvéglise, C., Oeser, B., Pearson, M., Poulain, J., Poussereau, N., Quesneville, H., Rascle, C., Schumacher, J., Ségurens, B., Sexton, A., Silva, E., Sirven, C., Soanes, D. M., Talbot, N. J., Templeton, M., Yandava, C., Yarden, O., Zeng, Q., Rollins, J. A., Lebrun, M. H., and Dickman, M. 2011. Genomic analysis of the necrotrophic fungal pathogens Sclerotinia sclerotiorum and Botrytis cinerea. PLoS Genet. 7:e1002230–e1002230

Cuppels, D. A. 1986. Generation and characterization of Tn5 insertion mutations in *Pseudomonas syringae* pv. *tomato*. Appl Environ Microbiol. 51:323–327

Derbyshire, M., Denton-Giles, M., Hegedus, D., Seifbarghi, S., Rollins, J., Kan, J. Van, Seidl, M. F., Faino, L., Mbengue, M., Navaud, O., Raffaele, S., Hammond-Kosack, K., Heard, S., and Oliver, R. 2017. The complete genome sequence of the phytopathogenic fungus *Sclerotinia sclerotiorum* reveals insights into the genome architecture of broad host range pathogens. Genome Biol Evol. 9:593–618

Dort, E. N., and Hamelin, R. C. 2024. Heterogeneity in establishment of polyethylene glycol-mediated plasmid transformations for five forest pathogenic *Phytophthora* species. PLoS One. 19:e0306158

Loper, J. E. 1987. Lack of evidence for in situ fluorescent pigment production by *Pseudomonas syringae* pv. *syringae* on bean leaf surfaces. Phytopathology. 77:1449

Perneel, M., Heyrman, J., Adiobo, A., De Maeyer, K., Raaijmakers, J. M., De Vos, P., and Höfte, M. 2007. Characterization of CMR5c and CMR12a, novel fluorescent *Pseudomonas* strains from the cocoyam rhizosphere with biocontrol activity. J Appl Microbiol. 103:1007–1020

Savchuk, S., and Dilantha Fernando, W. G. 2004. Effect of timing of application and population dynamics on the degree of biological control of *Sclerotinia sclerotiorum* by bacterial antagonists. FEMS Microbiol Ecol. 49:379–388

Sivachandra Kumar, N. T., Caudillo-Ruiz, K. B., Chatterton, S., and Banniza, S. 2021. Characterization of *Aphanomyces euteiches* pathotypes infecting peas in western Canada. Plant Dis. 105
